# The Domestic Staff

**Published:** 1908-08-22

**Authors:** 


### Nursing Administration.

### THE DOMESTIC STAFF.—I.

The Disappearance of the Scrubber.

The organisation of the domestic staff in institutions is undergoing a transition, in accordance with the changed conception of domestic service in the present day. It is, in fact, passing through a phase not dissimilar to that which nursing itself has undergone. Just as the old-time undisciplined nurse who came in, in the morning, with the milk, has given place to a new order, so the ragged regiment of scrubbers, who invaded the hospital precincts at given periods and laboured after their kind, is being replaced in modern hospitals by a neat array of young women, interested in their work, and regarding their future with hope. In the organisation of the domestic staff, the first step is to get rid of the outside worker. In theory, it is cheaper and vastly less troublesome to engage "hands" for the cleaning of stairs, ward-floors, passages, and dormitories. Such labour is a drug in the market, and no one feels any responsibility about the poor scrubber's character, or her prospects either in this life, or the world to come. But in reality it is an economy for which the hospital may very probably pay dear in the end. Who can picture the condition of the homes from which these scrubbers are drawn? The personal recommendation of a clergyman, which is about the high-water mark of respectability possible to attain, is a very slender security against the germs of measles or scarlet fever, and other less disastrous but more ordinary intruders being introduced into the wards and corridors of the hospital under cover of cleaning. This danger is a very real one, and many a mysterious outbreak of infectious disease might be directly traced to the scrubber, if any one thought her of sufficient importance to trouble about. This great drawback would suffice alone to discredit the services of outside workers. But there are others. The existence of a body of workers completely outside control, except in the matter of their particular spell of work is a perpetual source of annoyance to the matron. They may be first-rate workers, and yet by no means good examples in conversation and habits for the indoor domestic staff, on whose good tone so much of the comfort of the household must depend. It is a vain imagination to suppose that the indoor and outdoor staff can be completely severed from communication with each other. So a ready vehicle is at hand for stirring up insubordination, for engendering a lawless attitude towards hospital rules, and perhaps for leading young servants into graver danger. Again, the scrubber can hardly fail to be a cause of temptation in the kitchen. Either she has one of her meals on the premises, in which case it may be assumed that her entire board is provided by the institution, or she receives no board, in which case she is all the more a subject for kitchen bounties. There is every inducement to the cook to be open handed to this class of workers who are often worthy and very needy mothers of families, and it enormously increases the difficulties of the housekeeper if she has an army of hungry people always in the background to whose necessities she is not herself entirely callous. Now any leakage from the commissariat, even from good motives, constitutes a danger to hospital economy, and since both housekeepers and cooks are human and therefore merciful, it is better that the temptation to indiscriminate giving should be removed. Again, the work of keeping the building clean is one of very high importance when the work carried on in the establishment is considered. Scrubbing and cleaning used to be considered the very lowest and least skilled class of labour. But the knowledge of aseptics has changed this idea, and it is now understood that the process of keeping things clean is one which requires great accuracy, together with the use of proper materials and proper methods. How can the outside scrubber, whose only conception of cleaning steps is to slop filthy water over them, be instructed in the things which pertain to the science of cleanlines? She will regard precautions as fads, and evade rules whenever she can, the consequence being that the housekeeper, or sister, whose duty it may be to supervise the work, is conscious that her efforts to improve her workers and get them to take a pride in their work are like pouring water into a sieve. She has no hold on a constantly fluctuating body of women whose real interests lie, not in the performance of duty, but in getting home as soon as possible. Lastly, it may be contended that the hospital in using untrained workers for the cleaning of the establishment, and submitting to the fact that they must continue untrained, is missing a great opportunity. While every one in the institution is moving on, learning to do things well, then learning to teach others to do the same, it is a thousand pities this large section of workers should be excluded from the benefits of training. It is impossible to overrate the humanising and elevating influence which life in a well-ordered institution can wield over those who make up its units. For every scrubber employed, the hospital is losing the opportunity of moulding the character of an ignorant girl, who has no chance offered her of anything higher than muddling through her days unconscious of her powers. Every girl who tastes the pleasures of order, of duty happily discharged, and of well-earned promotion, is in a position to hand on to others the lessons she has learned herself. From an ineffectual waster, she is transformed into a skilled worker, and the world is so much the richer. There is every sign that we are only at the beginning of the revolution likely to be accomplished in the quality of the domestic servant of the future. A great responsibility rests upon employers to guide the latent ambitions and new energies of their servants in a healthy direction. And among the employers qualified to deal wisely with the material lying ready to hand, there are none who can offer better prospects of training and promotion than the matrons of institutions.

